# Freezing of gait and the effects of vibrotactile cueing in Parkinson’s disease–a study protocol of a cross-sectional multimodal brain imaging approach in virtual walking scenarios

**DOI:** 10.3389/fnins.2026.1872933

**Published:** 2026-07-09

**Authors:** Tim Göcking, Robert Stojan, Stefan A. Maas, Dieter F. Kutz, Claudia Voelcker-Rehage

**Affiliations:** 1 Department of Neuromotor Behavior and Exercise, Institute of Sport Science, University of Münster, Münster, Germany; 2 Movement Science Lab, Institute of Sports Science, Martin-Luther-Universität Halle-Wittenberg, Halle, Germany; 3 Joint Institute for Individualisation in a Changing Environment (JICE), University of Münster and Bielefeld University, Bielefeld, Germany

**Keywords:** electroencephalography, external cueing, functional near-infrared spectroscopy, gait disorders, neurorehabilitation, Parkinson, treadmill walking, virtual reality

## Abstract

**Background:**

Freezing of gait (FOG) is one of the most debilitating motor symptoms in Parkinson’s disease (PD), affecting a substantial proportion of people with PD. Numerous hypothetical mechanisms exist, attempting to explain FOG. Current evidence originated from stationary measures in lying positions, limiting the understanding of neurophysiological changes elicited by FOG episodes during regular locomotion. Neurophysiological aspects of FOG, especially in response to vibrotactile cueing, have been explored only by a few studies. To address this gap, the current study aims to investigate FOG episodes in people with PD and the impact of vibrotactile cueing while walking through virtual environments on a self-paced treadmill.

**Methods:**

Thirty people with Parkinson’s Disease and FOG will be recruited. They will undergo screening and familiarization before performing different walking tests in virtual reality using the Gait Real-time Analysis Interactive Lab (GRAIL). Participants will perform two experimental blocks, each consisting of four walking trials of approximately 10 min. A break of 10 to 30 min will be provided between blocks. Walking with or without vibrotactile cueing will be performed in alternation. During walking, electrocortical (EEG), hemodynamic (fNIRS), kinematic data (3D motion capture), and kinetic data (force plates) will be recorded. Data will be analyzed to investigate spatiotemporal and frequency characteristics of electrocortical and hemodynamic brain activity related to FOG and the impact of vibrotactile cueing on neural signatures and gait improvements in FOG.

**Discussion:**

Combining neurophysiological measurements of EEG and fNIRS paired with kinematics and kinetics will provide insights into the cortical and behavioral changes associated with FOG and vibrotactile cueing, to derive results that can be used for the design of intervention strategies for treating PD.

**Study protocol registration:**

https://drks.de/search/de/trial/DRKS00034584, identifier DRKS00034584.

## 1 Introduction

James Parkinson’s “An Essay on the Shaking Palsy” (1817) introduced the first formal medical description of Parkinson’s disease (PD), which today is one of the most common neurodegenerative disease worldwide. During recent decades, PD has been on the rise, with nearly 1 million people being affected by PD in the US alone, and this number is expected to rise to 1.2 million by 2030 (Marras et al., 2018). People with PD suffer from a wide range of motor symptoms, including tremors, muscle rigidity, bradykinesia, and postural instability (Giladi et al., 2001; Nutt et al., 2011). One of the most debilitating motor symptoms of the disease, however, is freezing of gait (FOG), of which approximately 60% of people with PD are suffering during the course of their disease (Macht et al., 2007; Zhang et al., 2021). FOG is described as a sudden and temporary inability to move forward. FOG manifests as either small steps in a forward shuffling motion, alternating trembling of the legs on site with a frequency of about 3–8 Hz, or akinesia (Hausdorff et al., 2003; Moore et al., 2008; Schaafsma et al., 2003). Typical triggers for FOG can be categorized as movement-related, cognition-related or emotion-related. Movement-related triggers of FOG include turnings, passing through (narrow) doorways or passages, and gait initiation (Cockx et al., 2023; Palmisano et al., 2022; Spildooren et al., 2019). Cognition and emotion-related FOG triggers, in turn, typically refer to situations with increased cognitive load or higher levels of anxiety (Martens et al., 2016; Pimenta et al., 2019; Spildooren et al., 2010). Unfortunately, the underlying neural mechanisms and pathways of FOG remain largely unclear, and current treatment options, such as dopaminergic medication and deep brain stimulation, often remain ineffective, particularly in resolving FOG. Given these challenges, so-called cueing strategies have emerged as effective alternatives to reduce FOG during daily life activities, among which vibrotactile cueing seems to be particularly promising. In this study, we will explore the neurophysiological correlates of FOG and assess the efficacy of vibrotactile cueing as a potential intervention to alleviate this debilitating symptom.

Recent reviews have summarized hypothetical neural pathways for the generation of FOG that include (i) interference between the basal ganglia (BG) and supplementary motor area (SMA), (ii) deficient crosstalk between BG, SMA and other cortical areas, (iii) interference between BG and frontal lobe, (iv) disruption along the dorsal visuomotor stream, and (v) uncoupling between SMA and motor cortex (Bardakan et al., 2022; Marquez et al., 2020; Nutt et al., 2011). Notably, these hypotheses and models share common but also unique (neural) pathways and mechanisms, supporting both the complexity and heterogeneity of the observed phenology of FOG, but also the idea of a common key process in the motor control circuit that ultimately triggers FOG. However, these neural mechanisms assumed to underlie FOG are predominantly based on stationary measurements, using gait imagery or stepping tasks in MRI laboratories. This contrasts with the dynamic nature of gait and FOG, creating a gap between experimental setups and real-world conditions. Recent advancements in ambulatory assessments, e.g., using mobile EEG / fNIRS during walking, now allow the investigation of FOG under more realistic, everyday-like conditions.

A few mobile EEG studies have investigated the electrocortical correlates associated with FOG in Timed Up-and-Go (TUG) tasks. They consistently found increased theta activity in right parietal and central brain regions during FOG episodes and during transition to FOG, suggesting an association of FOG with misguided integration of sensory information (Cao et al., 2021; Handojoseno et al., 2012, 2013; Shine et al., 2014). In addition, increased beta synchronization across the central cortex has been reported during FOG as well as an increase in beta activity across the parietal cortex along with changes in fronto-central beta coherence during transition to FOG (Handojoseno et al., 2015b; Shine et al., 2014). A surge in beta activation has been shown to be associated with motor symptoms in PD (Morelli and Summers, 2023) and is suggested to indicate impaired transmission of motor plans from frontal areas to the motor cortex (Shine et al., 2014). Additionally, investigating the differences between voluntary stopping and FOG, Cao et al. (2021) noted diminished power and different low-beta and delta power patterns in central regions during voluntary stopping compared to FOG (Cao et al., 2021). They also reported increased theta and alpha power over central and occipital regions during FOG compared to transition to FOG. Another study by Cockx et al. (2023) using fNIRS during walking showed a decrease in prefrontal cortex (PFC) activity during FOG compared to voluntary stopping (Cockx et al., 2024). Furthermore, they reported higher activation in the SMA and premotor cortex (PMC) in freezers compared to age-matched healthy controls during turning, stopping and walking through doorways. They emphasize the influence of the SMA in the pathophysiology of FOG and propose that FOG might emerge as a result of a disbalance between SMA and PFC. Moreover, connectivity analysis revealed increased coherence between central and frontal regions in the theta band during FOG, along with changes in theta synchronization during transition to FOG (Handojoseno et al., 2013; Shine et al., 2014). In this vein, Maidan et al. (2015) also reported an increased hemodynamic activation (fNIRS) in the frontal lobe before and during FOG (Maidan et al., 2015). However, these alterations were only observed in anticipated turns, indicating involvement of executive control mechanism in FOG; in line with the executive dysfunction hypothesis (Bardakan et al., 2022). Other important findings include intensified brain network synchronization with longer disease progression, which was consistently observed across various frequency bands (Asher et al., 2021). These results of increased coherence between frontal and central brain regions as well as increased network synchronization with increased disease severity potentially indicate the presence of compensatory neural mechanisms aimed at maintaining gait performance comparable to healthy controls (Poston et al., 2016).

In summary, previous studies have identified higher beta and theta power, higher synchronization and lower or higher hemodynamic brain activation in frontal and central regions during FOG or during transition to FOG. These findings are mainly based on modified versions of TUG tasks, providing limited transfer to more everyday-life walking conditions. Further, previous studies used either EEG or fNIRS, rather than employing a multimodal approach. The investigation of walking in VR and the simultaneous recording of EEG and fNIRS may increase the ecological validity and will allow to further understand the neurophysiology of FOG in PD. Understanding the neurophysiological correlates underlying FOG is crucial for the development and evaluation of effective intervention methods to ameliorate FOG in daily life.

Various approaches have been shown to be effective in counteracting the symptoms of PD, including FOG. Levodopa remains the most common medical treatment approach (LeWitt, 2015; Poewe et al., 2010; Warren Olanow et al., 2013), while more recent technological approaches, such as deep brain stimulation (DBS), have been used increasingly as an alternative therapeutic approach. However, both Levodopa treatment and DBS are associated with various side effects and health risks (e.g., invasive neurosurgery in DBS) (Fenoy and Simpson, 2014; Mosley and Akram, 2021). Therefore, exploring alternative approaches to enhance motor-related symptoms is an important step forward to complement current medical and neurosurgical therapies. Such alternatives include virtual and augmented reality-based intervention (Pokharel et al., 2026). For FOG, Cueing, described as temporal or spatial stimuli intended to facilitate and enhance motor execution (Nieuwboer et al., 2007), does not rely on drug intake or invasive operations. Cueing encompasses a range of modalities including auditory, visual, vibrotactile, and internal cueing (Cosentino et al., 2023). The most investigated cueing modalities to date are visual (presented as lines or patterns on the floor) and auditory cueing (e.g., rhythmic sounds or music). Various studies have shown the beneficial effects of these modalities in reducing FOG (Arias and Cudeiro, 2010; Barthel et al., 2018; Delval et al., 2014; Lirani-Silva et al., 2019; Velik et al., 2012; Wang et al., 2021). Neurophysiological evidence indicates that visual and auditory cueing induces alterations in the parietal and occipital brain regions in PD during cued walking (Stuart et al., 2021; Tosserams et al., 2022). However, the presentation of visual and auditory cues may distract users from relevant environmental information (e.g., warning ring of a passing bicycle, interpersonal interactions, or obstacles and uneven terrain). Additionally, users frequently report discomfort due to the visibility of these devices, as they attract attention to their condition, making them more easily recognized and potentially stigmatized in public.

Vibrotactile cueing, a less attention-demanding and discreet method using rhythmic vibrations as external cues, has been shown to improve balance (Ghoseiri et al., 2009) and reduce freezing episodes (Mancini et al., 2018; Rossi et al., 2020). Acute whole-body vibration has also been shown to modulate gait kinematics in people with PD, illustrating the broader potential of vibratory stimulation to influence gait (Oranges et al., 2025). So far, only one study by Stuart and Mancini (2020) investigated the neurophysiological correlates of tactile cueing by use of fNIRS (Stuart and Mancini, 2020). In this study, they reported favorable effects of open (constant rhythmic stimulation) and closed (stimulation based on individual movement) loop tactile cueing on usual walking and turning, but they did not report any changes in brain activation in response to vibrotactile cueing. However, cortical coverage was limited to the PFC. Although the PFC has been implicated in FOG in some studies mentioned above, the effects of vibrotactile cueing likely extend to other brain regions, particularly the parietal and central areas (e.g., SMA), which have also been reported to be involved in FOG (Cockx et al., 2024). Additionally, vibrotactile cueing may not just counteract disruptions in neural pathways associated with FOG, they may also contribute to alternative, compensatory neural pathways that indirectly help attenuate FOG. Given that limited evidence, the effects of cueing, in particular vibrotactile cueing, are not well understood to date.

In summary, research on FOG is lacking both behavioral and neural dimensions characterizing genuine cases of FOG, particularly in more ecologically valid contexts that are closer to everyday scenarios. Furthermore, there is a notable lack of studies investigating the behavioral and neural alterations associated with cueing, and in particular for vibrotactile cueing, one of the most promising modalities of current cueing strategies. In this study, we will therefore investigate the neural mechanisms underlying FOG and vibrotactile cueing in PD. We will take advantage of virtual-reality technology for investigating FOG under more everyday like but highly controlled experimental conditions. Although the main focus of this study is walking-related FOG, 360° turning is also included as an experimental condition, given its status of one of the most potent triggers of FOG and its ability to reliably elicit FOG in laboratory settings (Conde et al., 2023). Neurophysiological (EEG & fNIRS) and movement data (3D motion capture and ground reaction forces) during walking, turning and FOG episodes will be recorded and analyzed.

This research is motivated in particular by the limited evidence on the neural aspects of FOG and how effective countermeasures, such as vibrotactile cueing, can contribute to improvements of FOG related gait disturbances in everyday life. We will use a virtual walking scenario with automatic doorways to induce FOG to address three main research objectives. These objectives will focus on the neural mechanisms and pathways underlying FOG (RO1, RO2) and vibrotactile cueing (RO3). Our first research objective (RO1), aims to identify the cortical brain regions involved in FOG that show differential activation prior to the kinematic onset of FOG compared to normal, unimpaired walking. In the literature, the involvement of various brain regions such as frontal and central areas is reported (Asher et al., 2021; Cao et al., 2021; Handojoseno et al., 2015b; Shine et al., 2014). We expect involvement of similar brain regions (cf. perceptual dysfunction hypothesis (Bardakan et al., 2022)) in our virtual doorway scenario, but we also hypothesize differential activation based on the increased realism and environmental complexity achieved through the use of virtual reality. Second (RO2), we aim to delineate the neural signatures of induced FOG episodes, focusing on the temporal and frequency dynamics within the cortical areas of interest. Based on previous research we are expecting to see FOG specific changes in various frequency bands of interest, including alpha, beta and theta up to 5 s prior to FOG onset (Handojoseno et al., 2012, 2015b; Shine et al., 2014). To this end, we will compare FOG episodes vs. unimpaired walking through virtual doorways. Furthermore, we will analyze the connectivity of these dynamics across the brain regions involved. We expect to observe FOG-related changes in coherence, including increased theta coherence in fronto-central regions as reported previously (Shine et al., 2014).

Lastly (RO3), we aim to investigate whether and how vibrotactile cueing influences the neural patterns associated with induced FOG and contributes to its reduction. We expect that vibrotactile cueing will attenuate the neurophysiological changes linked to FOG (identified in RO2), so that prior to passing doorways, FOG-specific neural signatures will be less pronounced during cued walking than during non-cued walking. Thus, we will evaluate the differential activation patterns of non-cued vs. cued walking through virtual doorways and compare them to FOG specific activation. Additionally, we will assess reductions in the percentage of time spent frozen and analyze corresponding changes in gait kinematics and kinetics.

As part of this research plan, we will consider interindividual differences in FOG kinematics, kinetics, and neural correlates, as well as in the effects of vibrotactile cueing, such as by differential stages of disease, motor symptoms severity, gender, emotional well-being and other well-described moderating factors.

This clinical trial will be performed as a series of confirmatory cross-over studies with people with PD suffering from FOG under the three main research objectives specified above.

## 2 Materials and methods

### 2.1 Study setting

This study will be carried out at the laboratories of the Institute of Sport and Exercise Sciences of the University of Münster, Germany.

### 2.2 Eligibility criteria

Inclusion criteria are: (1) medical diagnosis of idiopathic Parkinson’s disease (Höglinger et al., 2023; Postuma et al., 2015) (2) age ≥ 18 years, (3) Montreal Cognitive Assessment (MoCA) (51) ( ≥ 18, (4) at least one daily freezing episode (reported by participants, assessed via telephone interviews), (5) normal or corrected-to-normal vision and (6) a stable medication for 4 weeks prior to inclusion (Ebersbach et al., 2010).

Exclusion criteria are: (7) previous history of traumatic brain injuries, (8) inability to stand or walk for at least 30 min without walking aids, (9) neuropsychiatric disorders (e.g., depression or anxiety), (10) any medication influencing the nervous system, (11) any severe comorbidities affecting gait, (12) any sensory impairments (e.g., due to polyneuropathy) hampering patients to perceive the vibration of the socks. All criteria are self-reported. Patient’s previous experience with cueing is not an exclusion criterion, but will be assessed as a potential confounding factor.

### 2.3 Intervention

Vibrotactile cueing will be applied to ameliorate FOG in PD. A customized vibrotactile cueing device, developed and provided by feelSpace (feelSpace GmbH, Osnabrück, Germany) that is based on a previous prototype as used by Koopman et al. (2019) and Klaver et al. (2023) will be utilized for the open-loop cueing conditions in this study (Klaver et al., 2023; Koopman et al., 2019). Eccentrically rotating mass motors (ERMs) will be used to generate rhythmic vibrations. The intensity of vibrations can be adjusted to the user’s preferences. The frequency ranges from 60 to 260 Hz and increases almost linearly with the voltage (0.6 V to 3.3 V). The vibration actuator will be driven by pulse width modulation and the intensity will be adjustable via API or an App. Increasing the voltage increases both the frequency and the amplitude. The actuator is located medially on the longitudinal arch of each foot, proximal to the calcaneus. The cueing intervention will be applied during walking. Participants will perform two experimental walking blocks. Between the two walking blocks, a break of 10 to 30 min will be provided to prevent exhaustion. Each block includes four walking trials within a virtual environment, each lasting approximately 10 min (cf. Virtual Walking Scenarios). Between trials, perceived physical and cognitive exhaustion are assessed. Cueing (intervention) and non-cueing (control condition) walking trials will be alternated within participants (with the starting condition randomly drawn) and balanced between participants (cf. Participant timeline). Alternation rather than randomization provides the advantage of ensuring balanced trial allocation, even if participants experience fatigue or withdraw earlier than expected. Despite efforts to mitigate fatigue through our experimental design, alternation further ensures an equal number of cued and non-cued trials, minimizing the risk of unequal trial distribution, and ensuring to have available at least one trial per condition. The experimental protocol will be terminated if a participant showed a total of 15 FOG events during non-cued walking (cf. Sample Size).

### 2.4 Outcomes

Primary outcomes are EEG electrocortical activity, i.e., spatial (RO1) and temporal (RO2) changes in different frequency bands. Outcomes will be compared between FOG and normal unimpaired walking, and between cued vs. non-cued walking (RO3) (cf. Statistical methods) to determine the effectiveness of the cueing intervention.

Secondary outcomes are fNIRS spatial (RO1) and temporal (RO2) FOG-related changes in hemodynamic activity, i.e., changes in oxygenated and deoxygenated hemoglobin, as well as changes in percentage time frozen and a pre-specified set of spatiotemporal gait parameters (step length, cadence, gait speed, and gait asymmetry and variability) together with ground reaction force measures. Outcomes will be compared between FOG and normal unimpaired walking, and between cued vs. non-cued walking (RO3) (cf. Statistical methods).

### 2.5 Participant timeline

Participants will attend two measurement days at the lab in order to reduce exhaustion and fatigue-related influences. The first lab visit will be used for screening and familiarization. An overview of the study will be provided and all necessary participation documents, such as informed consent, will be checked for completion. Subsequently, screening tests, including the Montreal Cognitive Assessment (MoCA) (Nasreddine et al., 2005), Movement Disorder Society Unified Parkinson’s Disease Rating Scale (MDS UPDRS) Part 3 (Goetz et al., 2008), Freiburg Visual Acuity test (Bach, 1996), and test of vibration sensitivity using a 128 Hz vibration fork (for further information cf. Screenings) will be administered. Head circumference will be measured to ensure proper fitting of the EEG-fNIRS cap. Body measurements will be taken in preparation for the motion capture at the second lab visit. These measurements include height, weight, leg length, knee width, ankle width, shoulder offset, elbow width, wrist width and hand thickness for each side of the body (Plug-in Gait Reference Guide - Nexus 2.12 Documentation, Vicon Documentation, VICON; Oxford, United Kingdom) (Vicon, 2023). Four reflective markers will be attached to the participants hip that are required to run the treadmill in self-paced mode. Participants will then put on a safety harness and commence a 15-minute familiarization period with the treadmill’s self-pace mode. Throughout this phase, participants will be instructed to adjust their walking speed to familiarize themselves with the self-paced feature within the virtual environment. Day 1 will proceed with regular medication. After screening and familiarization, participants will be provided with questionnaires through a digital platform (cf. Screenings). Once the participant has completed the questionnaire, the experimenter will verify that all questions have been answered without reviewing the content of the responses at this stage. All screening procedures and the familiarization on the treadmill will be performed and supervised by trained sport and exercise scientists with experiences in the field of movement science, motor control and neuroscience.

**FIGURE 1:**
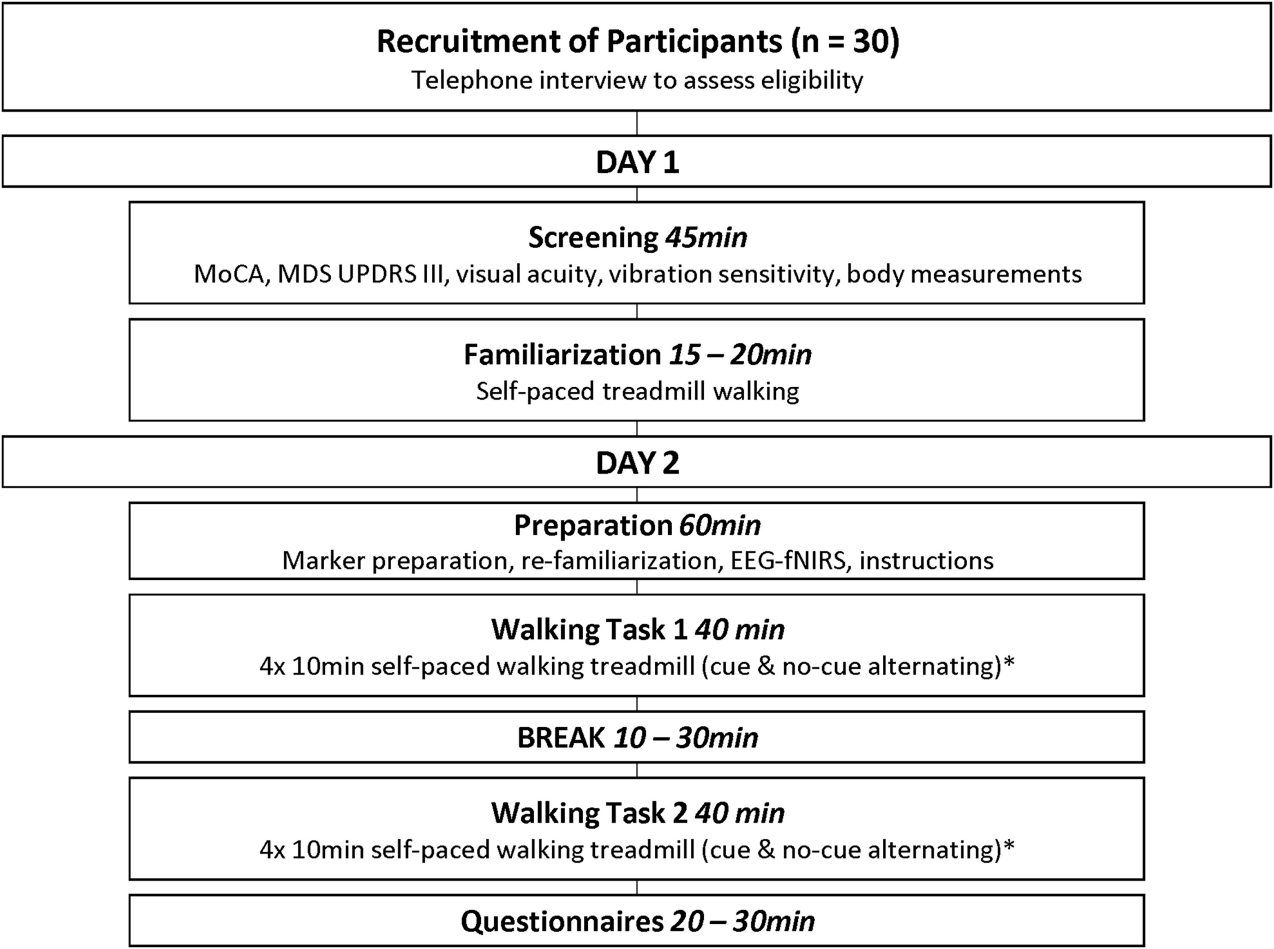
Flow chart of the participant timeline. This figure displays the procedure on both assessment days. MoCA, Montreal Cognitive Assessment; MDS UPDRS III, Part 3 of Movement Disorder Society Unified Parkinson’s Disease Rating Scale; EEG, Electroencephalography; fNIRS, Functional near-infrared spectroscopy. At the end of DAY 2 participants are asked to fill out further questionnaires to gather participant recommendations for the vibrotactile prototype used (cf. Screenings). *Each 10-min walking trial includes a 1-min baseline period immediately before trial onset.

On the second day, the session will start with participants being prepared for motion capture by applying reflective markers to the whole body and putting on a safety harness, followed by a short re-familiarization with the treadmill’s self-paced mode. Once comfortable, preparations for neurophysiological measurements will proceed. Participants will be seated and a cap, already equipped with 64 EEG electrodes, will be aligned on their head using anatomical landmarks (nasion, inion and preauricular points). Electrodes will be prepared with gel, followed by preparation of all optodes until adequate signal quality for both EEG and fNIRS is achieved (cf. sections Electroencephalography (EEG) and Functional near-infrared spectroscopy (fNIRS) for detailed information). The experimental procedure will begin with a detailed instruction of the virtual walking task and testing procedure. Participants are then performing a maximum of four walking trials, each lasting roughly 10 min. Before every trial, participants are asked to stand still for 1 min, which will serve as a baseline period for neurophysiological analyses. After each trial, a short break will be administered. If the total number of FOG episodes is below 15 and the patient is reporting a moderate amount of exertion, the next trial will be initiated. Exertion will be assessed using objective measures, including the Rate of Perceived Exhaustion Scale (Borg, 1982) and the Rating Scale Mental Effort (Zijlstra and Van Doorn, 1985). Participants will perform two blocks, each consisting of four walking trials. A break of 10 to 30 min will be provided between both blocks. Cueing and no-cueing will be administered in alternation. After 15 FOG events, the experiment will be stopped after completion of the current trial. The rationale for this design choice is to collect a sufficient number of FOG episodes, while not over exhausting participants. After the walking task 2, further questionnaires regarding the usability and feasibility of the prototype will be handed to the participants (cf. Screenings). For an overview of the whole procedure, see Figure 1. The neurophysiological preparations and measurements will be undertaken by the same personnel described above, who are trained in the preparation and application of various neuroimaging methods.

### 2.6 Sample size

An *a priori* sample size estimation was conducted using G*Power (v.3.1.9.4) for a linear multiple regression, fixed model with R^2^ increase, assuming an alpha level of 0.05, a power of 0.80, one primary predictor, and a total of five predictors (including covariates). We selected a moderate to large effect size of partial R^2^ = 0.25 (i.e., f^2^ = 0.33), informed in part from findings of Handojoseno et al. (2015b) on EEG electrocortical activity changes associated with transitions to FOG episodes (Handojoseno et al., 2015b). Additionally, as we plan to assess multiple FOG trials per person and condition (up to 15 trials) and analyze them using linear mixed effects models, which typically have higher statistical power than traditional aggregated mean analyses, we anticipate achieving sufficient power to detect effects even if they are somewhat smaller than initially estimated. The selection of 15 FOG episodes is driven by Cockx et al. (2024), who reported a median of 15 FOG episodes per participant and observed significant effects on neurophysiological changes, suggesting that up to 15 FOG episodes per participant may serve as a reasonable benchmark for achieving sufficient statistical power in the analyses (Cockx et al., 2024).

### 2.7 Recruitment

This study will be conducted at the University of Münster (Germany). The recruitment process will be carried out in cooperation with local Parkinson networks. Participants will be recruited by scientific staff via different channels including homepage advertisement and social media announcements, presentations at local Parkinson’s groups, personal contacts, local sport clubs and other non-profit associations as well as advertisements in local newspapers. Individuals expressing interest will be subject to a standardized telephone interview (10–15 min) to ascertain their eligibility based on predefined inclusion and exclusion criteria (cf. Eligibility Criteria). They will receive detailed information about the procedures of the study and must obtain medical approval before participation. Additionally, they are required to be accompanied by a healthy person when visiting our laboratory. Prior to enrollment, all participants will provide written consent to participate in this study.

### 2.8 Methods: data collection, management, and analysis

#### 2.8.1 Data collection methods

##### 2.8.1.1 Virtual walking scenarios

The virtual walking scenarios are implemented within the Gait Real-time Analysis Interactive Lab (GRAIL) (Motek Medical; the Netherlands). The GRAIL system integrates a 3D instrumented split-belt treadmill with two embedded force plates and a running surface of 1 m (width) x 2 m (length). Lateral handrails are attached to the treadmill, and two non-visible laser barriers are positioned at the front and rear ends for participant safety. Additionally, participants will be equipped with a safety harness connected to a ceiling load cable to mitigate the risk of falls. A stop button is in place on the experimenters’ control desk to promptly halt the treadmill in emergency situations. The GRAIL system also incorporates a marker-based, passive optical motion detection system (Vicon Motion Systems Ltd; Oxford, United Kingdom) (see Kinematics and Kinetics for further details) The virtual scenario is projected onto a semi-cylindrical 180° projection screen (2.4 m x 5 m) using three serially-connected RGB projectors. Another serially connected RGB projector projects onto the treadmill. To ensure accurate detection of visual stimulus presentations on the projection screen, a photodiode is fixed to the bottom center of the projection screen, imperceptible to participants. The photodiode detects any change in light intensity and therefore is used to detect an auxiliary projection (i.e., a white dot) that is synchronized with task-related stimuli displayed on the projection screen (Maas et al., 2024).

The treadmill is synchronized with the virtual environment and will operate in self-paced mode. The participants are able to walk at their preferred walking speed on the treadmill through the virtual environment. Participants are able to control their walking speed individually via their physical position on the treadmill using four reference markers on their hip. The reference point is placed 10 cm in front of the center of the treadmill. The front boundary is 55 cm in front of the center (45 cm from the reference point) and the rear boundary is 40 cm behind the center (50 cm from the reference point). Forward movements, i.e., moving the position of the markers in front of the reference point, accelerates the treadmill. Walking near the reference point maintains the current speed. When the markers are behind the reference point, the treadmill decelerates.

**FIGURE 2:**
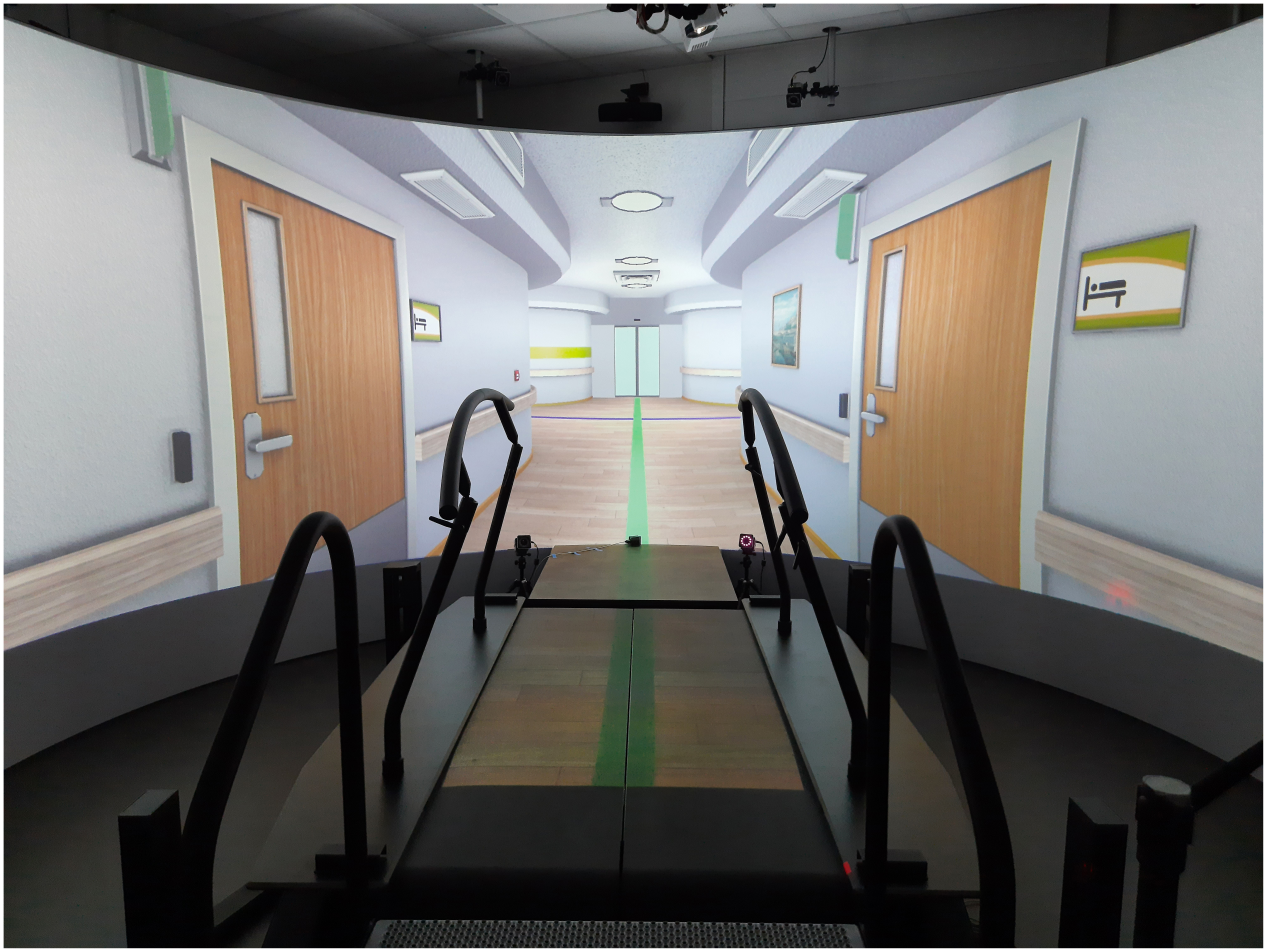
Virtual hospital scenario in Gait Real time Analysis Interactive Lab (GRAIL).

Virtual scenarios will be created with D-Flow (Motekforce Link, Amsterdam, the Netherlands). The virtual scenarios depict a hospital-like environment (see Figure 2) with an endless corridor with wooden flooring (brownish color) and a city scenario in a pedestrian zone. In the hospital scenario, which will be described here as an example, participants walk through a hospital-like corridor that contains visual representations of doors to other rooms (patient bedroom, dinner, medical specialists) to the right and left of the corridor in a well-lit environment. In addition, the scenario includes other smaller decorative objects (e.g., flower pots, benches, poster boards, lighting and ventilation, or trolleys) and occasional intersections with further decorative enrichments. These details aim at providing a more realistic and immersive virtual environment. The main aspect of the virtual hospital scenario are opaque automatic doorways. These doorways are used to mimic typical narrow doorways/passages in the real world that often cause FOG in PD (Cockx et al., 2023). The doors are designed as sliding doors with a motion sensor placed directly above the door. Behind some doors, objects such as a bench, potted flower, or trolley moving laterally toward the participant are presented randomly to introduce unpredictable events during the scenario. The automatic doors open bidirectionally at a speed of 0.3 m/s. They begin to open when the participant is positioned between 3.1–4 m (pseudorandomized, in steps of 0.1 m) in front of them. The doors open to a width ranging from 74 and 100 cm (pseudorandomized, in steps of 1 cm). The scenario contains 10 doors, each separated by approximately 30 m. A rotation symbol is projected onto the floor 8.5 m behind each door. The symbol consisted of two arrows pointing in the same direction and forming a circle, indicating that participants had to perform a 360° rotation. Once participants reached the floor-projected rotation cue and came to a stop, an enlarged version of the same cue was additionally displayed in the center of the screen. The required turn direction followed a fixed alternating sequence across the ten doors, always starting with a right turn at the first door, resulting in five right turns and five left turns.

The second scenario presents a virtual city scenario that portrays an urban environment with a pedestrian zone in which bicycles and pedestrians interact in virtual space. Direction and number of bicycles and pedestrians crossing or passing the participant will be varied, thus providing a dynamic and realistic representation of urban life in virtual space. The scenario includes a predefined sequence of 11 scripted events, which are distributed at regular intervals of approximately 30 meters along the walking path. These events consist of various interaction types, including pedestrians stepping out of buildings and crossing or walking alongside the participant’s path, passing maneuvers, stationary individuals initiating movement as the participant approaches, irregular or nonlinear walking patterns and bicycle crossings. After about 215 meters, the environment transitions to a section featuring architectural arches. Participants continue walking under the arches while additional event types occur.

##### 2.8.1.2 Electroencephalography (EEG)

Brain activity will be captured during walking utilizing a mobile EEG system (LiveAmp, Brain Products GmbH, Gilchingen, Germany) equipped with 64 gel-based active Ag/AgCl electrodes (actiCAP slim electrodes, Brain Products GmbH, Gilchingen, Germany) positioned according to the modified 10-10 system (Nuwer et al., 1998). The following electrode positions will be used: Fp1, Fp2, AF7, AF3, AFz, AF4, AF8, F7, F5, F3, F1, Fz, F2, F4, F6, F8, FT9, FT7, FC5, FC3, FC1, FCz, FC2, FC4, FC6, FT8, FT10, T7, C5, C3, C1, Cz, C2, C4, C6, T8, TP9, TP7, CP5, CP3, CP1, CPz, CP2, CP4, CP6, TP8, TP10, P7, P5, P3, P1, Pz, P2, P4, P6, P8, PO7, PO3, POz, PO4, PO8, O1, Oz, O2. Electrode FPz will be used as a ground electrode. Impedances will be kept below 25 kOhm, which has been shown to be eligible for active electrodes (Laszlo et al., 2014). EEG signals will be acquired with a sampling rate of 500 Hz. Data will be recorded during the walking tasks on day 2 (cf. Participant timeline). For each walking trial, a separate EEG recording will be made. Both amplifiers of EEG and fNIRS will be carried within a vest worn by the participant. Efficient cable management ensures full freedom of movement for the upper and lower extremities as well as for the head. Synchronized timestamps for the beginning and end of the trials as well as for the approaching doors will be send via LSL (cf. System integration – fNIRS, EEG and GRAIL). Timestamps for FOG episodes will be extracted from the video data and subsequently incorporated into the EEG data (cf. FOG detection).

##### 2.8.1.3 Functional near-infrared spectroscopy (fNIRS)

**FIGURE 3:**
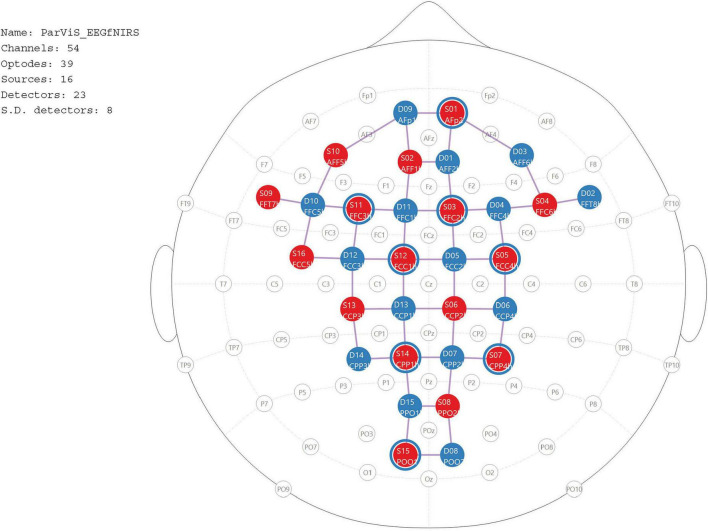
Probe setup. Bi-hemispheric setup covering whole head with 64 EEG electrodes (white) and with 16 sources (red), 15 long-distance detectors (blue), and 8 short channel detectors (blue circle outlines); Graphics were created with NIRSite from NIRx Medical Technologies (courtesy of NIRx Medical Technologies LLC; https://nirx.net).

Hemodynamic activation during walking will be recorded using two 8 x 8 mobile NIRSports 2.0 systems (NIRx Medical Technologies, Glen Head, NY, USA) and Aurora recording Software (NIRx Medical Technologies, Glen Head, NY, USA). The two systems will be configured in a single-subject tandem setup, including 32 dual-tip optodes. The setup consists of 16 light emitters and 15 long-distance detectors spaced approximately 3–4 cm apart (long channels), adapted to the 10-05 system standards (Oostenveld and Praamstra, 2001). Additionally, eight short channels detectors will be utilized with a source-detector distance of about 1 cm. This distance allows for assessment of extracerebral physiological and mechanical artifacts, while not capturing brain-related hemodynamic activity (Yücel et al., 2015). The sensor arrangement will create 46 long-distance channels focusing on several key areas of interest (Cockx et al., 2024), spanning frontal, central, parietal, and occipital regions across both hemispheres (cf. Figure 3). For different head circumferences, four different cap sizes (Bach, 1996; Borg, 1982; Koopman et al., 2019), 60 cm; NIRScap, EASYCAP GmbH, Herrsching, Germany) will be used to ensure a comfortable fit and to consider the anatomical differences in the brain. The sources emit infrared light pulses at two wavelengths (760 nm and 850 nm) with a modulation frequency of 5.086 Hz (continuous wave fNIRS). Further, sources are time-multiplexed to prevent crosstalk between channels. All optodes are shielded from external light sources (e.g., screen light) by an opaque cover. To ensure that participants have sufficient freedom of movement, all optode cables and the amplifier are firmly stowed and held in a small backpack on the participants’ backs. Further experimental and statistical measures will be implemented, including a high number of FOG trials (up to 15 FOG trials per person) and advanced statistical approaches (multilevel models) to improve the robustness of our findings (Ayaz et al., 2022; Yücel et al., 2021).

##### 2.8.1.4 Kinematics and kinetics

Kinematic and kinetic data will be captured using VICON Nexus 2.12 (VICON; Oxford, United Kingdom) software, two independent ground reaction force plates, fixed 10 infrared cameras (VICON VERO; VICON Motion Systems Ltd; Oxford, United Kingdom), and reflective markers. The cameras will be calibrated before each recording session and capture videos at a frequency of 100 Hz. Reflective markers will be positioned according to the Plug-in-Gait full body marker set, with a total of 39 markers for each participant (Vicon, 2023). The following angles will be calculated: elbow angles, head angles, neck angles, shoulder angles, spine angles, thorax angles, wrist angles, ankle angles, hip angles, knee angles, and the pelvis angles. The joint angles will be calculated by the VICON Nexus 2.12. software using previously collected body measurements (VICON; Oxford, United Kingdom). The full-body joint angles provide information about the movement of the whole body, including head and trunk movement, that is used for signal-quality control and artifact handling of the EEG and fNIRS data. They are not treated as outcome measures (cf. Outcomes). The measuring system utilizes the two independent force plates to record 3D ground reaction forces at a frequency of 1000 Hz for each leg separately (Motek Medical; the Netherlands). The VICON Nexus software will export ground reaction force data, raw marker trajectories and joint angles. The raw marker trajectories will be exported as XYZ coordinates. The joint angles will be exported as either vector, relative, or absolute angles, depending on the joint. The force data will be exported for each force plate individually, including location and force vector, as well as a combined value.

##### 2.8.1.5 FOG detection

Participants will be recorded by three cameras (Basler ace acA720-290gm, Basler AG) operating at 50 fps. Two cameras are positioned laterally to capture movements in the sagittal plane, and one is positioned behind the participant to record movements in the frontal plane. Two independent experts will assess these videos for FOG episodes and annotate detected episodes using the video annotation tool ELAN, following the updated guidelines of Gilat et al. (2026) (The Language Archive, 2024). This procedure represents the gold standard in the field for detecting and classifying FOG (Moore et al., 2013). Additionally, each annotated episode will be classified by the motor context at its onset as either walking-FOG (forward locomotion, including doorway passage and straight walking) or turning-FOG (360° turning). This classification is based on the video and the synchronized task markers. The percentage of time spent frozen will be calculated by dividing the duration of the episodes annotated as FOG by the duration of the gait tasks.

##### 2.8.1.6 System integration—fNIRS, EEG and GRAIL

Synchronized timestamps are integral across all systems for seamless coherence and precision. Lab streaming layer (LSL) (Kothe et al., 2019) will be used to synchronize the different measurement systems, all sending their data via a local network generated by a Raspberry Pi (Raspberry Pi Foundation, United Kingdom). This setup was validated experimentally by our workgroup (Maas et al., 2024).

##### 2.8.1.7 Screenings

Initial screenings will be conducted via telephone, including the prescribed eligibility criteria. On site, further screenings are undertaken by scientific staff: Part 3 of the MDS-Unified Parkinson’s Disease Rating Scale (MDS-UPDRS) (Goetz et al., 2008), the Montreal Cognitive Assessment (MoCA) (Nasreddine et al., 2005) and Freiburg Visual Acuity Test (Bach, 1996). The vibratory sensitivity will be measured with a 128 Hz Rydel-Seiffer tuning fork at the medial longitudinal arch of the foot, such as the medial cuneiform bone (Buchman et al., 2009; Hilz et al., 1998; Martina et al., 1998; Strzalkowski et al., 2015; Wolf et al., 2022).

Additionally, participants will be prompted to provide further behavioral and demographic information via questionnaire, encompassing details such as age, disease onset, medication type and dosage, social and cognitive activities of daily living as well as already existing experiences with cueing strategies or devices.

Further, participants will be asked to fill in the following questionnaire: Parkinson’s Disease Questionnaire (PDQ-39) (Jenkinson et al., 1997), New Freezing of Gait Questionnaire (NFOG) (Seuthe et al., 2021) and Godin-Shephard leisure-time Physical Activity Questionnaire (Godin, 2011). All questionnaires will be administered using the SoSci Survey platform (Leiner, 2024). The collected data will be used to provide a comprehensive description of the sample. Participants will have the option to complete the questionnaires on day 1. On day 2, all given answers will be checked for misunderstandings and clarified with the participant if necessary. In addition, further questionnaires will be handed to the participants on day 2 after all walking trials: System Usability Scale (Brooke, 1996) and a custom made questionnaire designed to gather participant recommendations for the vibrotactile prototype. All data will be assessed by experienced sport and exercise scientists.

#### 2.8.2 Data preprocessing and analysis

##### 2.8.2.1 EEG

EEG-Data will be preprocessed in MATLAB (MathWorks, Massachusetts, USA) using the BeMoBIL Pipeline, following the authors’ recommendations (Klug et al., 2022). Non-experimental segments will be removed using the synchronized start and end markers. The data will be resampled to 250 Hz and frequency specific noise will be removed using Zapline-plus with automatic noise frequency detection (Klug and Kloosterman, 2022). Bad channels (EEG channels with abnormal signal quality, indicated by low correlation with estimates derived from neighboring scalp electrodes) will be identified and interpolated. The data will be re-referenced to the average. For independent component analysis, the data will be high-pass filtered at 1.5 Hz for AMICA decomposition. The resulting ICs will be classified using the IClabel “lite” as classifier, with components showing a probability > 70% for muscle, eye, heart, line or channel noise being removed. The cleaned data will then be high-pass filtered at 0.2 Hz and subsequently be imported into MNE Python for further analysis (Gramfort et al., 2013). Morlet Wavelet analysis will be applied to extract the time-frequency components of interest within a 5-second time window preceding the FOG marker, effectively capturing the transition from regular locomotion to FOG. These extracted components will then be subjected to further statistical analysis.

##### 2.8.2.2 fNIRS

Data analysis will be conducted using commonly used software, such as MNE Python (Gramfort et al., 2013). All raw data will be converted to the standardized *.snirf format, recognized for fNIRS data. Each dataset will be carefully reviewed to remove channels that do not meet the quality standards based on a signal quality index. This will include discarding channels that show a consistently low signal across significant portions of the recording sessions. Motion artifacts will be identified and corrected using typical correction methods such as spline interpolation and wavelet-based corrections, with specific thresholds for standard deviation and amplitude. Raw optical densities will be converted into changes in hemoglobin concentrations using the modified Beer-Lambert Law. This step will incorporate age and wavelength-specific differential pathlength factors to account for individual differences in optical properties. Slower systemic changes, often introduced by physical activities or head movements, will be mitigated by bandpass filtering and regressing out signals from short channels and specific head movement metrics. Finally, the signals from each channel will be normalized (z-transformed) to enable averaging and epoching across channels within the same cortical regions. This step ensures that the data are normally distributed, facilitating subsequent analysis. Spatial and temporal characteristics of the hemodynamic signal (e.g., peak amplitude, latency, etc.) related to FOG will be extracted and subjected to further statistical analysis.

##### 2.8.2.3 Kinematics and kinetics

All gait parameters will be derived using customized scripts in commonly used software, such as MATLAB (MathWorks, Massachusetts, USA). In particular, heel-strike and toe-off times will be determined. Heel-strikes will be identified by analyzing the z-axis (up and down) of the heel markers, while toe-offs will be identified using the z-axis of the toe markers. The gait events’ exact timing will be calculated by identifying the corresponding inclines and declines in the force curves, utilizing the higher frequency of the force plates. The exact timing of the heel-strikes and toe-offs will then be used to calculate temporal aspects of the gait, like stance and swing time. Heel-strikes also represent the start and end of each gait cycle, therefore giving reverence points in continuous timeseries like joint angles. The computer controlling the treadmill will provide an export specifying the current velocity and distance covered at any given moment. Other gait parameters will be computed on a stride-by-stride basis using common operational definitions and will be calculated and exported for further analysis.

#### 2.8.3 Data management

All collected data will be pseudonymized using coding lists. These lists are located in a locked cupboard within a locked room at the University of Münster. The coding lists will be destroyed after completion of data acquisition, ensuring the data will be anonymized from that point forward. Anonymized data will be stored for a minimum of 10 years on password protected servers of the University of Münster, limited to authorized researchers directly involved in data acquisition and analysis.

#### 2.8.4 Statistical methods

To address our main research objectives, we will employ linear mixed effects models for our analyses. These models are well-suited for handling repeated measures and allow us to take advantage of the multi-trial data structure, while also accounting for both within-subject and between-subject variability. FOG episodes will be analyzed separately by motor context, distinguishing walking-FOG (forward locomotion, including doorway passage and straight walking) from turning-FOG (360° turning). Each FOG type will be compared against its corresponding unimpaired movement (Handojoseno et al., 2015a). The number of FOG episodes per type and participant will be reported.

First, we will identify cortical brain regions showing differential activation prior to the kinematic onset of FOG. Dependent variables include spatiotemporal characteristics of electrocortical and also hemodynamic brain activity that will be compared between FOG episodes and the corresponding period of unimpaired walking or turning. Second, we aim to delineate the neural signatures of FOG episodes, focusing on the temporal and frequency dynamics within the cortical areas of interest as defined by the first part of our analysis. This step includes analyzing the coherence of the neural dynamics across the brain regions involved. Lastly, we will examine if and how vibrotactile cueing may alter the neural signatures of FOG described above and whether it contributes to improved gait behavior when walking through virtual environments. This analysis will also focus on the reduction of the percentage time frozen, changes in kinematic and kinetic aspects of gait. All analyses will include relevant covariates, such as disease stage, motor symptom severity, prior experience with cueing and random effects accounting for inter-subject variability, and to investigate interindividual differences. To address the large number of comparisons across modalities, all analyses will include appropriate correction for multiple comparisons to control the rate of false-positive findings, using established methods such as Bonferroni correction or cluster-based permutation testing (Maris and Oostenveld, 2007).

### 2.9 Methods: monitoring

#### 2.9.1 Data monitoring

No data monitoring committee is required as the measurements and interventions are conducted by qualified, impartial project employees. The employees also monitor participants for symptoms such as vertigo, confusion, pain, or any other symptoms during both the screening and familiarization on day one, and the walking tasks on day two. They are prepared to intervene if necessary.

#### 2.9.2 Harms

For measurements on day 2, participants are required to refrain from taking medication for 12 h. That refrainment could exacerbate the symptoms of PD, similar to regular dose failures, and which may last until the day after. Physical and cognitive exhaustions of participants is monitored continuously between trials (cf. Participant timeline) and various safety measures are applied during testing (cf. Virtual Walking Scenarios). After the walking tests, participants are asked to take their usual medication and have a break.

#### 2.9.3 Auditing

None.

## 3 Discussion

With this study we aim to investigate the neural mechanisms and pathways underlying FOG (RO1, RO2) and the effects of vibrotactile cueing (RO3). To achieve this, we will examine the neurophysiological correlates of FOG using ambulatory EEG and fNIRS measurements in virtual walking environments. Participants will walk on a treadmill while immersed in these environments, allowing us to study the neurophysiological changes related to FOG episodes. In addition, a vibrotactile cueing device will be used aiming to improve FOG. Thus, we will compare cued versus non-cued walking and investigate its influence on the frequency of FOG episodes and the associated changes in brain activation. For RO1, we anticipate that our study will reveal distinct neurophysiological correlates of FOG in PD. Building on previous research that has identified key brain regions, such as the frontal and central areas (Asher et al., 2021; Cao et al., 2021; Handojoseno et al., 2015b; Shine et al., 2014), and various hypothetical neural mechanisms (e.g., executive dysfunction and interference model (Bardakan et al., 2022)) that seek to explain the pathophysiology of FOG, we expect to observe similar findings while also gaining additional insights through our novel and more ecologically valid experimental design. For RO2, we specifically expect to observe neurophysiological changes across various frequency bands of interest, including alpha, beta, and theta, occurring up to 5 s prior to the observable behavioral manifestations of FOG episodes when approaching virtual automatic doorways (Handojoseno et al., 2012, 2013, 2015b). For RO3, we hypothesize that vibrotactile cueing will reduce the occurrence of FOG episodes through mitigation of the neurophysiological changes linked to FOG (cf. RO2). Specifically, we expect that the neural signatures related to FOG will be less pronounced during cued walking compared to non-cued walking when approaching doorways.

By understanding the neural mechanisms underlying FOG episodes and the potential attenuating effects of vibrotactile cueing, we hope to contribute to the development of effective intervention methods for managing FOG in PD. The high temporal resolution of neurophysiological changes preceding FOG episodes may provide new insights about the timing of cueing devices to be effective and to potentially improving their efficacy. It might further give insights into how to design other appropriate intervention strategies, such as individualized cueing, to reduce FOG. Enhancing the effectiveness of cueing devices and developing alternative intervention strategies has the potential to refine the gait pattern in PD and thus improve patient mobility. This improvement in mobility can lead to more social interaction and therefore a better quality of life and psychological well-being (Lee and Choi, 2020). In addition, by facilitating a more stable gait, the number of falls in PD patients may be reduced, which in turn could lead to a reduction in hospitalization rates and associated healthcare costs.

## 4 Ethics and dissemination

### 4.1 Research ethics approval

This study was approved by the local Ethics Committee of the University of Münster, Faculty of Psychology and Sport and Exercise Sciences, Münster (protocol code: 2024-12-RS-Breaking the Freeze – Behavioral and Neural Aspects of Freezing of Gait and Vibrotactile Cueing in Parkinson’s Disease”; date of approval: 04.05.2024).

### 4.2 Protocol amendments

Protocol amendments will be approved by the local Ethics Committee of the University of Münster, Faculty of Psychology and Sport and Exercise Sciences, Münster.

### 4.3 Consent or assent

Members of the study team will introduce the study to the patients and provide all relevant information regarding the trial. Patients will receive an informed consent documentation, including a detailed overview of the study objectives, methods of data collection and data protection. Researchers will be available to answer any questions. Written informed consent will be obtained from all patients. The informed consent will also clearly state that participants can withdraw from the study at any time without any negative consequences.

### 4.4 Confidentiality

Collected data will be pseudonymized, securely stored, and anonymized after data acquisition. Anonymized data will be stored for at least 10 years on password-protected servers to which only authorized researchers have access (cf. Data Management).

### 4.5 Access to data

Access to anonymized data will be limited to authorized researchers directly involved in data acquisition and analysis. The data and code that support the findings of this study are available upon reasonable request from the corresponding author, C.V.-R.

### 4.6 Ancillary and post-trial care

Participants must be accompanied by a healthy person, and both the participant and their companion are covered by a travel insurance policy for their journey to and from the laboratory.

### 4.7 Dissemination policy

The findings of this study will be shared publicly through various channels: publication in peer-reviewed scientific journals related to the study’s topic, oral presentations at specialized conferences, regularly scheduled network meetings of Parkinson Münsterland +, and our social media channels and department homepage.
